# Antimony susceptibility of *Leishmania* isolates collected over a 30-year period in Algeria

**DOI:** 10.1371/journal.pntd.0006310

**Published:** 2018-03-21

**Authors:** Naouel Eddaikra, Khatima Ait-Oudhia, Ihcen Kherrachi, Bruno Oury, Farida Moulti-Mati, Razika Benikhlef, Zoubir Harrat, Denis Sereno

**Affiliations:** 1 Laboratory of Eco-epidemiology Parasitic Population Genetics, Pasteur Institute of Algiers, Algiers, Algeria; 2 IRD, Univ Montpellier, MIVEGEC, Montpellier, France; 3 Laboratory of Analytic Biochemistry and Biotechnology, Univ Mouloud Mammeri, Tizi Ouzou, Algeria; 4 National Veterinary School, Algiers, Algeria; 5 IRD, Univ Montpellier, InterTryp, Montpellier, France; Saudi Ministry of Health, SAUDI ARABIA

## Abstract

**Background:**

In Algeria, the treatment of visceral and cutaneous leishmanioses (VL and CL) has been and continues to be based on antimony-containing drugs. It is suspected that high drug selective pressure might favor the emergence of chemoresistant parasites. Although treatment failure is frequently reported during antimonial therapy of both CL and VL, antimonial resistance has never been thoroughly investigated in Algeria. Determining the level of antimonial susceptibility, amongst *Leishmania* transmitted in Algeria, is of great importance for the development of public health policies.

**Methodology/Principal findings:**

Within the framework of the knowledge about the epidemiology of VL and CL amassed during the last 30 years, we sampled *Leishmania* isolates to determine their susceptibility to antimony. We analyzed a total of 106 isolates including 88 isolates collected between 1976 and 2013 in Algeria from humans, dogs, rodents, and phlebotomines and 18 collected from dogs in France. All the Algerian isolates were collected in 14 localities where leishmaniasis is endemic. The 50% inhibitory concentrations (IC50) of potassium antimony tartrate (the trivalent form of antimony, Sb(III)) and sodium stibogluconate (the pentavalent form of antimony, Sb(V)) were determined in promastigotes and intramacrophage amastigotes, respectively. The epidemiological cutoff (ECOFF) that allowed us to differentiate between *Leishmania* species causing cutaneous or visceral leishmaniases that were susceptible (S+) or insusceptible (S-) to the trivalent form of antimony was determined. The computed IC50 cutoff values were 23.83 μg/mL and 15.91 μg/mL for VL and CL, respectively. We report a trend of increasing antimony susceptibility in VL isolates during the 30-year period. In contrast, an increase in the frequency of S- phenotypes in isolates causing CL was observed during the same period. In our study, the emergence of S- phenotypes correlates with the inclusion of *L*. *killicki* (syn: *L*. *tropica*) isolates that cause cutaneous leishmaniasis and that have emerged in Algeria during the last decade.

**Conclusion/Significance:**

Our results provide insight into the spatiotemporal dynamics of *Leishmania* antimony susceptibility in Algeria. We highlight the need for the future implementation of an effective methodology to determine the antimony susceptibility status of *Leishmania* isolates to detect the emergence of and prevent the dissemination of drug-resistant strains.

## Author summary

In Algeria, the treatment of visceral and cutaneous leishmaniases (VL and CL) has been and continues to be based on antimony-containing drugs. It is suspected that high drug selective pressure might favor the emergence of chemoresistant parasites. Within the framework of knowledge about the epidemiology of VL and CL amassed during the last 30 years, we sampled *Leishmania* isolates to analyze their susceptibility to antimony. The epidemiological cutoff (ECOFF) that allowed us to differentiate between *Leishmania* species causing cutaneous or visceral leishmanioses that were susceptible (S+) or insusceptible (S-) to the trivalent form of antimony was determined. The computed IC50 cutoff values were 23.83 μg/mL and 15.91 μg/mL for VL and CL, respectively. We report a trend of increasing antimony susceptibility of VL isolates during the 30-year period. In contrast, an increase in the frequency of S- phenotypes in isolates causing CL was observed during the same period. The emergence of these S- phenotypes appears to correlate with the appearance of cutaneous leishmaniasis caused by *L*. *killicki* (syn: *L*. *tropica*). We highlight the need for the future implementation of an effective methodology to determine the antimony resistance status of *Leishmania* isolates to detect the emergence of and prevent the dissemination of drug-resistant srains.

## Introduction

Leishmaniasis are vector-borne diseases caused by obligate parasites from the genus *Leishmania* (*Trypanosomatida*: *Trypanosomatidae*). This genus is subdivided into two major phylogenetic lineages: *Euleishmania* and *Paraleishmania* [1]. There are fifty-four named species of *Leishmania* (without considering synonyms) and approximately twenty species that are pathogenic to humans [2,3] (www.leishmania.ird.fr). These diseases are endemic in large areas of the tropics and subtropics and in the Mediterranean basin, globally spanning more than 98 countries and territories. There are approximately 350 million people at risk for leishmanioses and approximately 12 million cases worldwide, with an estimated annual incidence of 0.7–1.2 million cases of CL and 0.2–0.4 million cases of VL [4].

Antimony (Sb)-containing compounds currently represent the first-line drugs for the treatment of all forms of leishmaniosis in most parts of the world, mainly for economic reasons. The antileishmanial and antitrypanosomal activity of Sb was investigated in the 1910s [5,6], and Sb continues to be used in antiprotozoal drugs [7]. In 1997, the first indications of therapeutic failure linked to *Leishmania* drug resistance were reported in the northern Bihar province of India [8–10].

In Algeria, cutaneous and visceral leishmanioses constitute a public health concern. Algeria ranks second, after Afghanistan, for the incidence of cutaneous leishmanioses [11]. The visceral form, recorded in humid and sub-humid regions located in the north of the country, is caused by *Leishmania infantum*, with dogs as the main reservoir and *Phlebotomus perniciosus* or *P*. *longicuspis* acting as vectors [12–16]. This disease affects mainly children under 5 years of age [17–20]. *L*. *major*, *L*. *infantum* and *L*. *killicki* (syn *L*. *tropica*) are the identified causative agents of the cutaneous forms of the disease in Algeria [21–23]. Cases of cutaneous leishmaniosis caused by *L*. *major* are widely distributed across the arid zones of the southern part of Algeria [24]. The identified mammalian reservoir hosts of *L*. *major* are wild rodents, including *Psammomys obesus* and *Meriones shawi* [25,26]. Its proven vector is *Phlebotomus papatasi* [27]. In the northern humid and sub-humid parts of the country, *L*. *infantum* is responsible for the sporadic form of CL. The main vector is *P*. *perfilewi*, and the reservoir hosts are dogs [28,29]. In addition, an outbreak of cutaneous leishmaniosis caused by *L*. *killicki* (syn *L*. *tropica*) and transmitted by *Phlebotomus sergenti* was detected in 2005 in the province of Ghardaia [21,30,31]. Canine leishmaniosis (CanL) caused by *L*. *infantum* is a zoonotic disease that affects millions of dogs [32]. The clinical features of the disease vary from subclinical, self-limiting infections to fatal disease [33]. In Algeria six zymodemes have been found to infect dogs, but the most frequently recorded is *L*. *infantum*, belonging to the MON-1 zymodeme [34,35]. In Algeria, pentavalent antimonials are the standard treatments for all forms of leishmaniosis. Treatment failures are frequently reported during antimonial therapy of both CL and VL [19,20,36,37]. The causes underlying treatment failures are multifactorial and interplay with certain host factors (e.g., host genetics and immunological responses), treatment features (e.g., drug quality, compliance with therapy), and the intrinsic drug susceptibility/resistance of *Leishmania* strains or species [8,38–41]. These multiple factors make the analysis of antimony treatment failures and antimony resistance challenging.

In Algeria, as in most of the 98 countries where leishmanioses represent a major threat, no epidemiological information related to the antimony resistance status of *Leishmania* parasites is currently available. In the present study, we determined the antimony susceptibility profile of various *Leishmania* isolates belonging to species endemic in Algeria. We discuss the essential information obtained and its interpretation in the framework of public health interventions against leishmanioses in Algeria.

## Materials and methods

### Study design

We sought to determine the antimony susceptibility of *Leishmania* isolates for the most prevalent species transmitted in Algeria. The isolates used in the study are responsible for the visceral form of the disease (*L*. *infantum*) or for cutaneous forms (*L*. *infantum*, *L*. *major*, and *L*. *killicki* syn *L*. *tropica*). They were collected from patients (*Homo sapiens*), mammalian reservoirs (*Canis familiaris* and *Meriones shawi*) and phlebotomine sandflies (*Phlebotomus perniciosus* and *Phlebotomus papatasi*) (Table 1).

**Table 1:** Identification of the 106 *Leishmania* isolates, collected from humans, dogs, rodents and phlebotomines and used in the study.

Hosts	Disease	Species	Number	Country	Zymodeme	Date of sampling
**Human**	VL	*L*. *infantum*	19	Algeria	MON-1 & MON-80	1980–2005
	VL	*NK*	02	Algeria	ND	2010–2011
**Human**	CL	*L*. *infantum*	06 03	Algeria Algeria	MON-24 & MON-80 ND	2009–2013
**Human**	CL	*L*. *major*	20	Algeria	MON-25	2009–2013
**Human**	CL	*L*. *killicki* (*L*. *tropica*)	08	Algeria	MON 301	2005–2011
**Dog**	CanL	*L*. *infantum*	24 16	Algeria France	MON-1 & MON-281 MON-1	2006–2008 1974–2006
**Rodent**	-	*L*. *major*	03	Algeria	MON-25	1982–2006
**Phlebotomine** ***P*. *papatasi***	-	*L*. *major*	03	Algeria	MON-25	1989
**Phlebotomine** ***P*. *perniciosus***	-	*L*. *infantum*	02	France	MON 1	1984
**Total**			108			

ND: not determined

**Fig 1:**
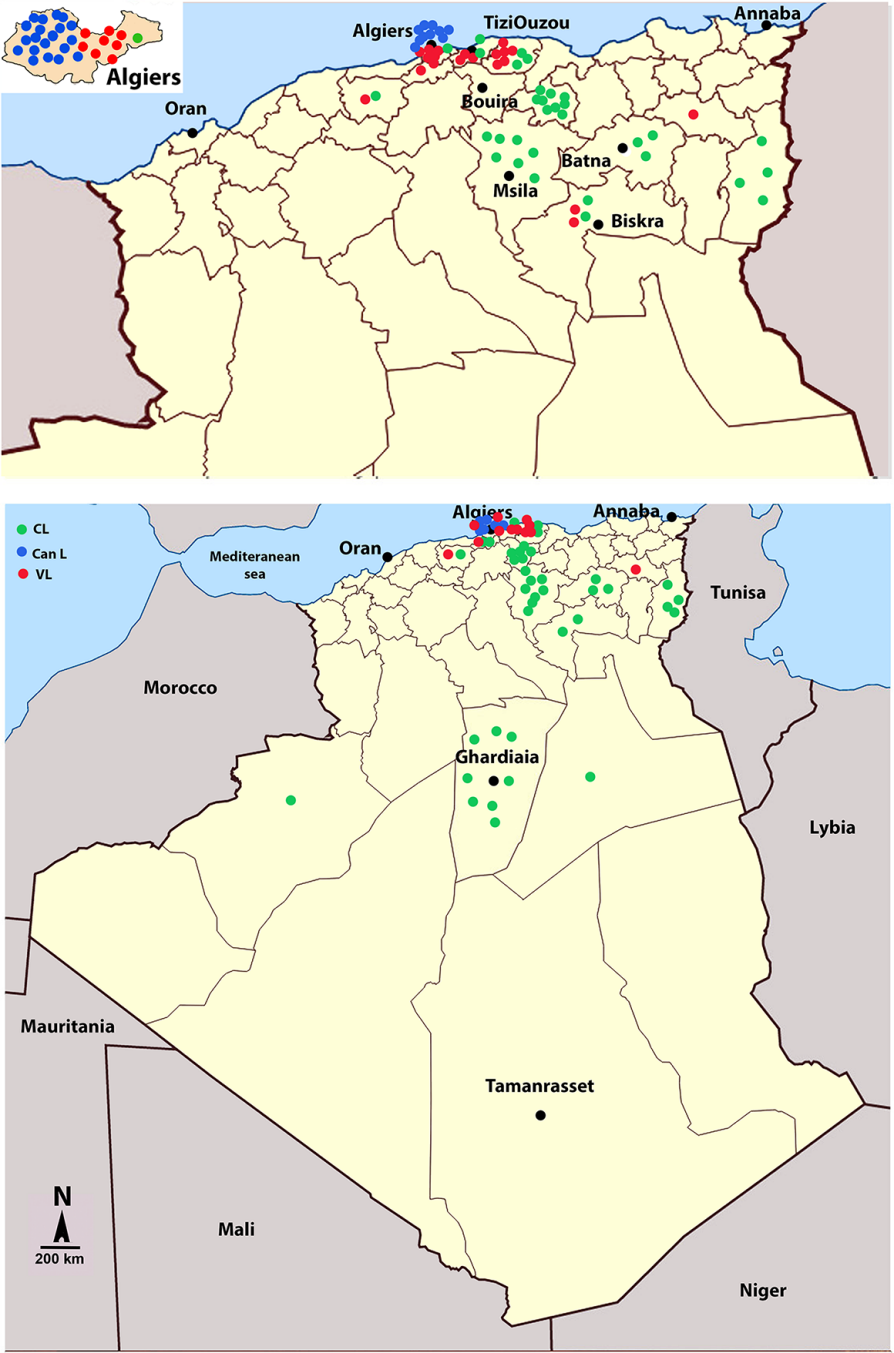
Geographic origin of *Leishmania* isolates and strains used in the study. Parasites were sampled from dogs (CanL) or from human individuals with visceral (VL) or cutaneous leishmaniases (CL). Geographical origin of the isolates (**A**). Focus on northern territories of Algeria and of the Alggier region (insert) (**B**).

Samples were collected during clinical and community-based studies conducted at the Pasteur Institute of Algiers since 1980. They now constitute a documented collection of *Leishmania* clinical isolates that were previously typed at the zymodeme level [29,35,42]. These resources, in addition to demographic and other data (clinical patterns, disease incidence, geographical locations of cases, etc.) of the endemic foci studied, were exploited to examine the epidemiological associations. The geographical distribution of samples is given (Fig 1A & 1B).

### Study area description

Four ecological zones of unequal sizes compose Algeria. The northern Tell region has a Mediterranean climate characterized by warm, dry summers and cool, wet winters (total annual rainfall is from 400 to 1000 mm). Natural forests are concentrated in a 100- to 200-km-wide belt in the northern part of the country that is more humid (rainfall = 200–400 mm). The Sahara Desert is an arid (total annual rainfall >130 mm), windy area with large daily temperature differences. The Hoggar massif (7% of the country) lies south of the Sahara. It is a succession of high-altitude desertic areas rising in terraces to an altitude of 2900 m. Administratively, Algeria is divided into 48 departments.

### Algerian leishmaniasis data compilation and analysis

In Algeria, leishmanioses have been reportable diseases since 1980. The National Public Health Institute (INSP) gathers and centralizes information about cases of cutaneous and visceral leishmanioses from the 48 departments throughout the country. Data from the official case notifications were compiled from the epidemiological surveillance database of the INSP for a 30-year period from 1984 to 2013. They result from passive monitoring based on the notifications registered by the epidemiological services of health sectors in the territories (local public health facilities, public hospitals, and university hospitals). Private physicians are required to notify the INSP about VL cases.

### Collection of *Leishmania* strains

*L*. *major*, *L*. *killicki* and *L*. *infantum* parasites were isolated from patients and rodents by the medical staff of the national reference centers for leishmanioses (Pasteur Institute of Algiers). They were then cryopreserved in liquid nitrogen until they were thawed for antimony susceptibility testing. The 24 *Leishmania* isolates from naturally infected dogs were collected by veterinary practitioners in various regions of Algiers between November 2006 and June 2008 (Fig 1, see insert). The popliteal lymph nodes were aspirated from symptomatic dogs, and a skin biopsy was taken from dogs with inoculation chancres on the nose. In addition, French *Leishmania* isolates were selected from the cryobank of the National Reference Centre for Leishmanioses in Montpellier, according to their sampling date and location. We choose to restrict our study to two canine leishmaniosis endemic foci, Béziers and Montpellier. The origins and descriptions of the isolates used in this study are compiled in Table 1. Prior to conducting the drug susceptibility assays, the species identity was re-confirmed by isoenzyme electrophoresis characterization and PCR-RFLP, as described elsewhere [43–45]. The susceptibility of the *Leishmania* strains to antimony was determined *in vitro* within four passages from their thawing. A total of 106 isolates were studied, 88 collected in Algeria and 18 in France (S1 Table and S1 Fig). Among them, 37 were responsible for human cutaneous leishmanioses, and 21 were responsible for the visceral form. In addition, the susceptibility to antimony was determined in isolates collected from reservoirs or vectors, including 24 from dogs, 3 from sandflies and 3 from rodents. Isolates that originated in France were collected from dogs and sandflies. A cloned Sb(III)-resistant *L*. *infantum* strain (ITMAP263-R/Sb3-120R) and its sensitive counterpart (ITMAP263) were used as controls [46]. Synthetic graphs showing the distributions of *L*. *major*, *L*. *infantum*, *L*. *tropica* (*L*. *killicki*) in the various selected localities and according to the clinic and the period of sampling are presented in S1 Fig.

### Strain typing

Isoenzymatic characterization was performed with starch gel electrophoresis according to Rioux (43) using the following 15 enzyme systems: malate dehydrogenase (MDH), EC 1.1.1.37; malic enzyme (ME), EC 1.1.1.40; isocitrate dehydrogenase (ICD), EC 1.1.1.42; 6-phosphogluconate dehydrogenase (PGD), EC 1.1.1.44; glucose-6-phosphate dehydrogenase (G6PD), EC 1.1.1.49; glutamate dehydrogenase (GLUD), EC 1.4.1.3; NADH diaphorase (DIA), EC 1.6.2.2; purine nucleoside phosphorylases 1 (NP1), EC 2.4.2.1; purine nucleoside phosphorylases 2 (NP2) EC 2.4.2; glutamate-oxaloacetate transaminases (GOT1 and GOT2), EC 2.6.1.1; phosphoglucomutase (PGM), EC 5.4.2.2; fumarate hydratase (FH), EC 4.2.1.2; mannose-phosphate isomerase (MPI), EC 5.3.1.8; and glucose phosphate isomerase (GPI), EC 5.3.1.9. In the PCR-RFLP analysis, the entire internal transcribed spacer (ITS) in the ribosomal operon was amplified using LITSR (5′-CTGGATCATTTTCCGATG-3′) and LITSV (5′-ACACTCAGGTCTGTAAAC-3′) primers [45]. A 10-μL aliquot from the amplification product of the entire ITS region was then digested with HaeIII for 2 h at 37°C. The restriction fragments were then separated by electrophoresis on a 2% agarose gel for 2–4 h in 1x Tris-borate/EDTA (90 mM Tris-borate/0.2 mM EDTA) buffer. The restriction fragments were visualized under UV light after ethidium bromide staining.

### Antimony susceptibility determination

A maximum of 5 passages in liquid culture media were performed before promastigote susceptibility assays. Promastigotes were maintained at 26°C in SDM-79 medium [47–48] supplemented with 10% fetal calf serum (FCS), 50 μg/mL porcine hemin, 2 mM glutamine, 100 IU/mL penicillin, and 100 μsg/mL streptomycin.

Promastigote susceptibility assay: growth inhibition of *Leishmania* treated with the trivalent form of antimony, Sb(III), was determined by flow cytometry. Parasites were inoculated into 96-well plates at 10^6^ parasites/mL in 100 μl of SDM-79 with fetal calf serum. Sb(III) antimony was added at final concentrations of 0.3, 0.6, 1.2, 2.4, 4.8, 9.6, 19.2, 38.4, and 76.8 μg/ml. After 3 days of culturing, 5 μL of the cell culture were taken and diluted into 500 μL of PBS (pH 7.2). Propidium iodide was added at a final concentration of 1 μg/mL, and the cells were counted by flow cytometry (FACScalibur; Becton Dickinson) for 52 sec. Fluorescence gated according to forward and side light scatter was collected and displayed using a logarithmic amplification (FL2–FL3). The statistic quadrant allowed us to estimate the number of viable parasites. The parasite density was determined using standard curves, where parasite concentrations were plotted as a function of the mean number of cells counted after 52 sec. The fifty percent inhibitory concentration (IC50) value was determined with GraphPad Prism 6.0 software (GraphPad Software, La Jolla, CA, USA) using a sigmoidal dose-response (variable slope) curve fitting equation that allowed the calculation of the concentration that inhibited the growth by 50% (IC_50_). The results are expressed as the means of three independent experiments performed in duplicate.

Intramacrophagic amastigote susceptibility assay: the effect of Glucantime on *Leishmania* growth in a human leukemia monocyte cell line (THP-1 cells) was evaluated according to the method previously described [49]. THP-1 cells were cultured in RPMI 1640 medium supplemented with 10% FCS, 2 mM glutamine, 100 IU/mL penicillin, and 100 μg/mL streptomycin. THP-1 monocytes in the log phase of growth were differentiated into macrophages by incubation for 2 days in medium containing 20 ng/mL phorbol-myristate acetate. Macrophages were then infected with stationary-phase *L*. *infantum* promastigotes at a parasite:macrophage ratio of 5:1 for 4 h, at 37°C with 5% CO2. After removing non internalized parasites by washings, cultures were incubated in the presence of Glucantime for 5 days at 37°C in an atmosphere enriched with 5% CO2. Medium added with Glucantime was renewed after 72 h incubation. Infected cells were then washed, fixed with methanol and stained with Giemsa. The parasitic index PI was then calculated as follows: PI(%) = (percentage of infected macrophages × number of intracellular parasites/macrophage in treated wells)/(percentage of infected macrophages × number of intracellular parasites/macrophage in untreated wells) × 100. Then IC_50_ value was determined.

### Statistical analysis and susceptibility/epidemiological cutoff determination

Statistical analysis: Data were compiled in Microsoft Excel and analyzed using Prism 5 (GraphPad, Inc). We computed standardised incidence ratios (SIR) that represent the ratio of the reported rate for CL and VL to the age-adjusted expected rate in the general population. This allow comparison between provinces taking the national average incidence rate as a reference, during the three decades studied. *Leishmania* isolates from each region were selected using a simple random sampling technique. Human leishmanioses (VL & CL) incidence were plotted on a map with an Open Source Geographic Information System QGIS 2.14. We used the website http://download.geofabrik.de/africa/algeria-latest-free.shp.zip to download the administrative regions of Algeria and we obtained the georeferenced data on the cities of Algeria from http://dateandtime.info. For leishmania antimony susceptibility comparison, a one-factor ANOVA was performed to determine a significant difference between groups with differences in susceptibility to Sb(III) and Sb(V) between Algerian isolates according to clinical forms, *Leishmania* species, zymodems and spatial geographic distributions. ANOVA analysis was followed by Kruskal-Wallis (KW) tests and Dunn’s post-test for multiple comparisons. Differences in the susceptibility to Sb(III) and Sb(V) among VL and CL isolates during two 10-years-time periods were analyzed using the Mann-Whitney non-parametric test. Correlation of susceptibility to Sb(III) and Sb(V) was estimated with the Spearman test. P values <0.05 were considered significant.

Cutoff determination: The cutoff defining sensitive (S+) and non sensitive (S-) promastigotes populations for Sb(III) was determined with the help of a web application: cutoff finder analysis using R version 2.15.0 (2012-03-30) (http://molpath.charite.de/cutoff/) [50]. The cutoff for Sb(V) susceptibility was directly obtained by interpolation of the Sb(III)cutoff values.

### Ethics statement

Clearance for the study was obtained from the Ministry of Health (Direction of Prevention) of Algeria. Informed consent was not obtained for our study because the data were analyzed anonymously. *Leishmania* isolates from human (*Homo sapiens*) were obtained from routine clinical works. *Leishmania* isolates from dogs (*Canis familiaris*) rodents (*Meriones shawi*) or sandflies (*Phlebotomus papatasi* and *Phlebotomus perniciosus*) were obtained from routine epidemiological studies in endemic areas. This study was exempted of an ethical approval.

## Results

### Leishmanioses in Algeria: Analysis of data collected over a 30-year period

A schematic representation of the three geographic areas of Algeria and the total number of leishmanioses cases (CL and VL) recorded in those areas over the three consecutive decades studied are given (S2 Fig).

**Fig 2:**
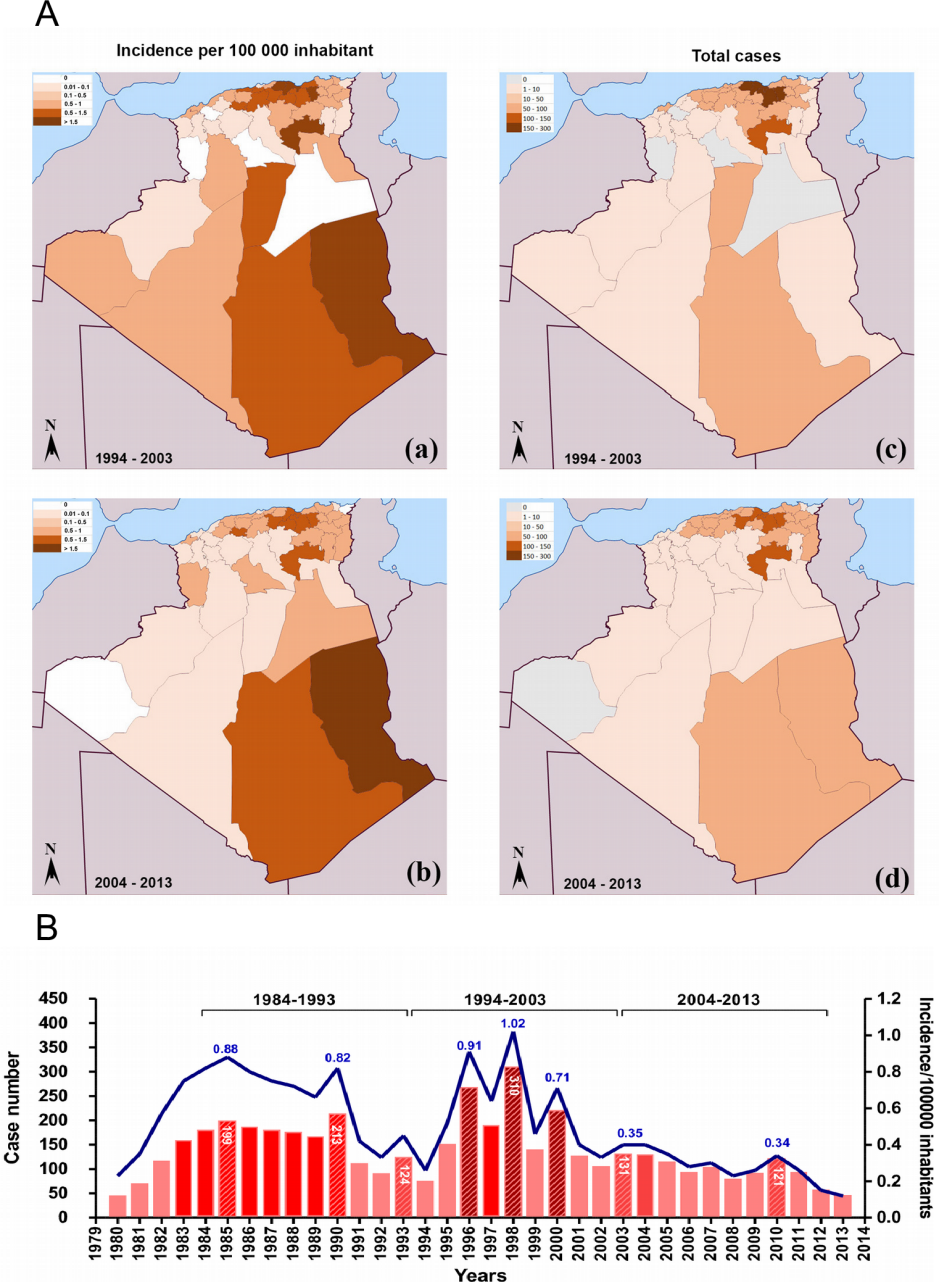
Records of visceral leishmaniasis (VL) incidence in Algeria from 1994 to 2013. Geographic origin of the reported cases of VL in Algeria (A), from 1994 to 2003 (a) and from 2004 to 2013 (b). Evolution of the reported cases and incidence in Algeria from 1982 to 2013 (B). Data were compiled from the Algerian National Public Health Institute (INSP).

Between 1984 and 2013, the greatest number of case notifications of VL occurred in 1998, when 310 cases were reported. During this period, the highest incidence of VL was recorded in the Tell area (Fig 2A and S2 Fig). An overall increase in the notification of VL cases was recorded between 1994 and 2003, followed by a decrease during the subsequent decade (Fig 2B). The calculated average annual incidence rate was 0.68 cases per 100,000 inhabitants in 1969, 0.88 cases in 1985 and 1.02 cases in 1998. In 2010, a rebound was recorded, with 121 case notifications. Nevertheless, the incidence could be considered low, with 0.34 cases per 100,000 inhabitants (Fig 2A and 2B). When the geographical distribution of VL cases was assessed, we observed a decrease in disease incidence in the historical foci of Bouira and Tizi Ouzou, that decrease from 1.55 and 0.79 cases per 100,000 inhabitants during the 1994 to 2003 period in Tizi Ouzou and Bouira respectiveley to 1.43 and 0.28 cases during the 2004 to 2013 period. In other regions, the situation has changed even more radically, with a total absence of notifications in recent years. This situation was found mainly in the northwest and the south. However, in Tamanrasset, located in Southeast Algeria, a resurgence in case notifications was observed.

**Fig 3:**
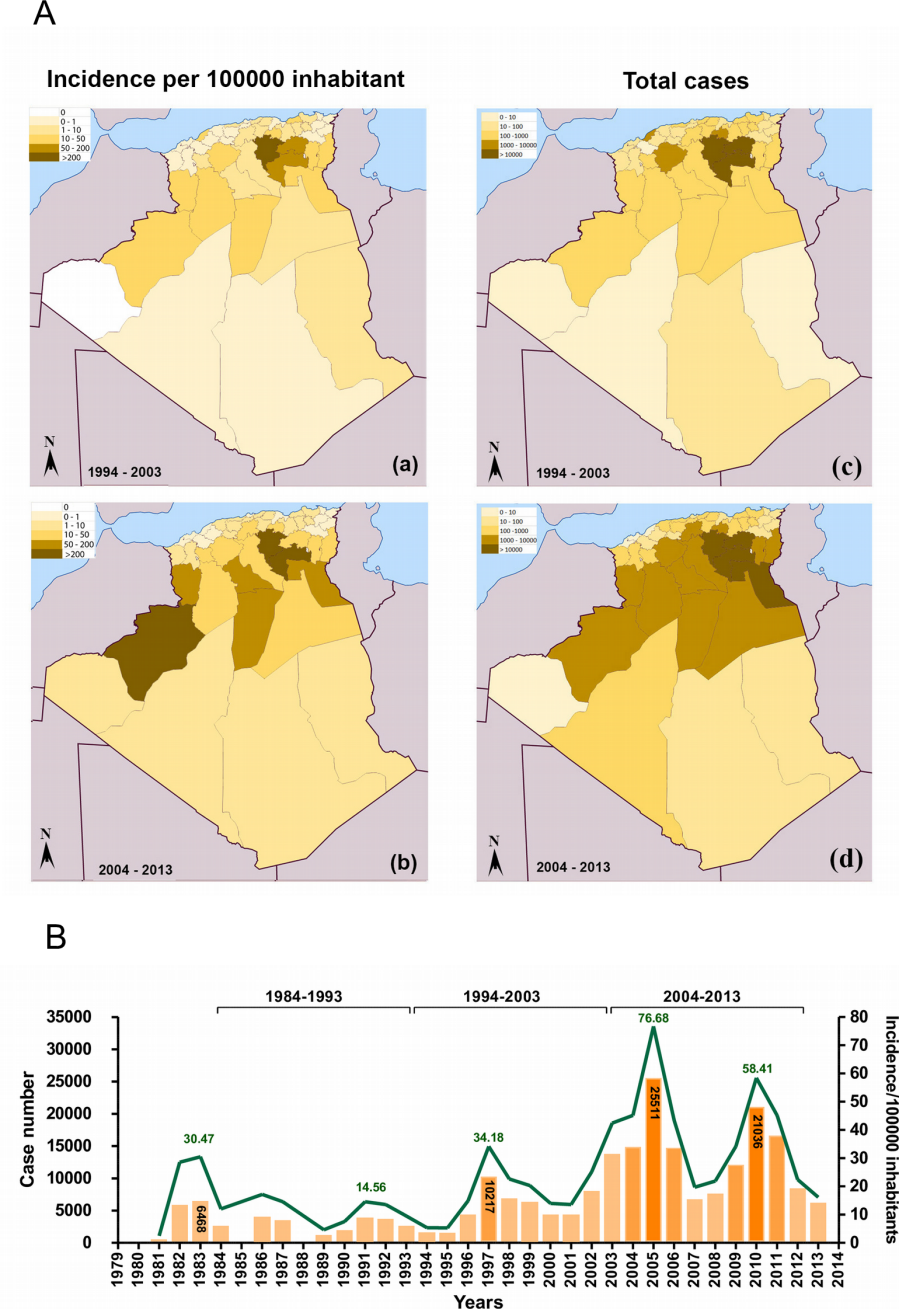
Records of cutaneous leishmaniases (CL) incidence in Algeria from 1994 to 2013. Geographic origin of the reported cases of CL in Algeria (A), from 1994 to 2003 (a) and from 2004 to 2013 (b). Evolution of the reported cases and Incidence in Algeria from 1982 to 2013 (B). Data were compiled from the National Public Health Institute (INSP).

Overall, CL have a 30-fold higher incidence rate than the visceral form. The most affected areas are located on the steppes in the sub-Saharan areas of Algeria. Data collected from the monthly epidemiologic report published by the INSP showed that between 2004 and 2013, the disease extended over the southern and northern portions of the country (Fig 3A). Most of the CL notified cases came from the departments of Batna, Biskra and M'sila [24]. Several peaks of CL have occurred over the last 30 years, namely, in 1983, 1986, 1997, 2005 and 2010 (Fig 3B). The greatest number of cases was recorded in 2005, when there were more than 25,000. In 1991, an incidence rate of 15 cases per 100,000 inhabitants was recorded, and this incidence rate remained almost unchanged five years later (14.44 cases per 100,000 inhabitants), but it reached 34.18 cases per 100,000 inhabitants in 1997, 76.68 cases in 2005 and 58.41 cases in 2010 (Fig 3B). Thus, the analyzed 30-year period demonstrates an overall decrease in the total number of VL cases in all endemic regions of Algeria and the opposite trend for CL cases.

### Antimony susceptibility of promastigote and amastigote forms of *Leishmania*: Determination of epidemiological cutoff values

**Fig 4:**
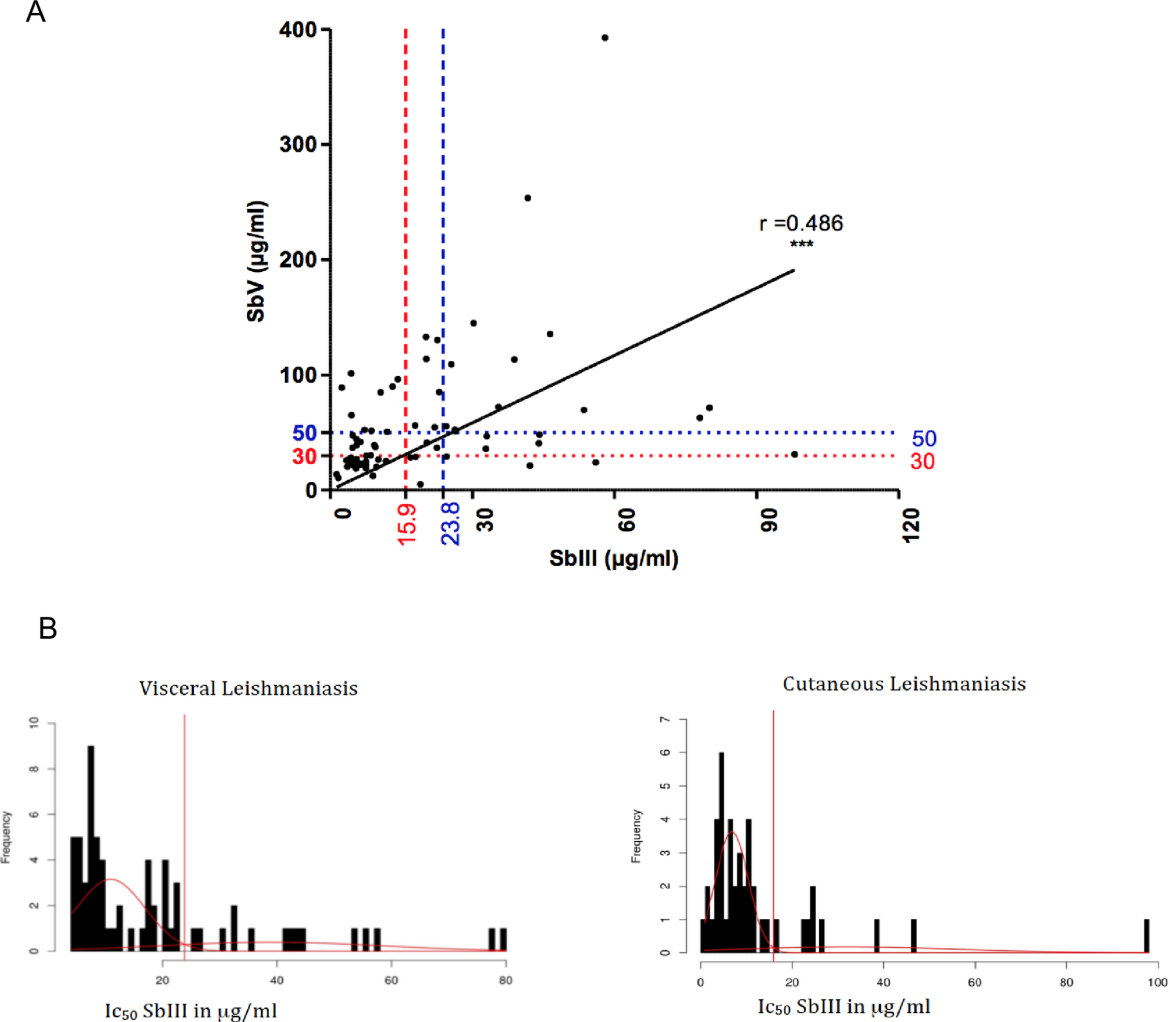
Correlation between susceptibilities of promastigotes and intracellular amastigotes to Sb(III) and Sb(V), respectively. Correlation between Sb(III) and Sb(V) susceptibilities (A). Dached vertical line materialize Sb(III)-ECOFF values for VL (blue) and CL (red) isolates. Horizontal lines represent cut-off (ECOFF) values for Sb(V) which are deduced via projection of Sb(III)-ECOFF values on the Sb(V) IC50 ordonate axis according to the regression curve. Determination of the epidemiological cutoff values for Sb(III) (Sb(III)-ECOFF) of isolates responsible for VL and CL (B).

The correlation between the susceptibility of promastigotes and amastigotes to Sb(III) and Sb(V) was investigated with a panel of 73 of the 88 isolates that were representative of the diversity of all the *Leishmania* species with public health impacts in Algeria. The susceptibility of the promastigote forms to Sb(III) reflected the susceptibility of intracellular amastigotes to Sb(V) (Spearman’s correlation, P<0.0001, r = 0.486). This finding highlights the predictive value of the Sb(III) susceptibility of promastigotes for the Sb(V) susceptibility status of intracellular amastigotes (Fig 4A). We then determined the IC50 ECOFF value for Sb(III) (Sb(III)-ECOFF). The cutoff values for VL and CL isolates were determined with a method based on the data distribution. The output of the analysis was visualized as a histogram in which the cutoff value was defined at the peak (Fig 4B). The ECOFF values were determined according to the clinical forms described as CL (caused by some *L*. *infantum* variants, *L*. *major*, and *L*. *killicki* (syn *L*. *tropica*)) or VL (caused by *L*. *infantum*). The computed Sb(III)-ECOFF against promastigotes was 15.91 μg/mL among the species responsible for the VL forms and 23.83 μg/mL among the species responsible for the CL forms (Fig 4B). The Sb(V)-ECOFF was then deduced via a direct projection of the Sb(III)-ECOFF values on the ordinate axis via the regression line (Fig 4A). Using this approach, the Sb(V)-ECOFF values of 50 μg/mL (blue dashed line) and 30 μg/mL (red dashed line) were deduced for the *Leishmania* species responsible for CL and VL, respectively.

### Determination of the antimony susceptibility of various *Leishmania* species responsible for distinct clinical forms of leishmaniases

**Fig 5:**
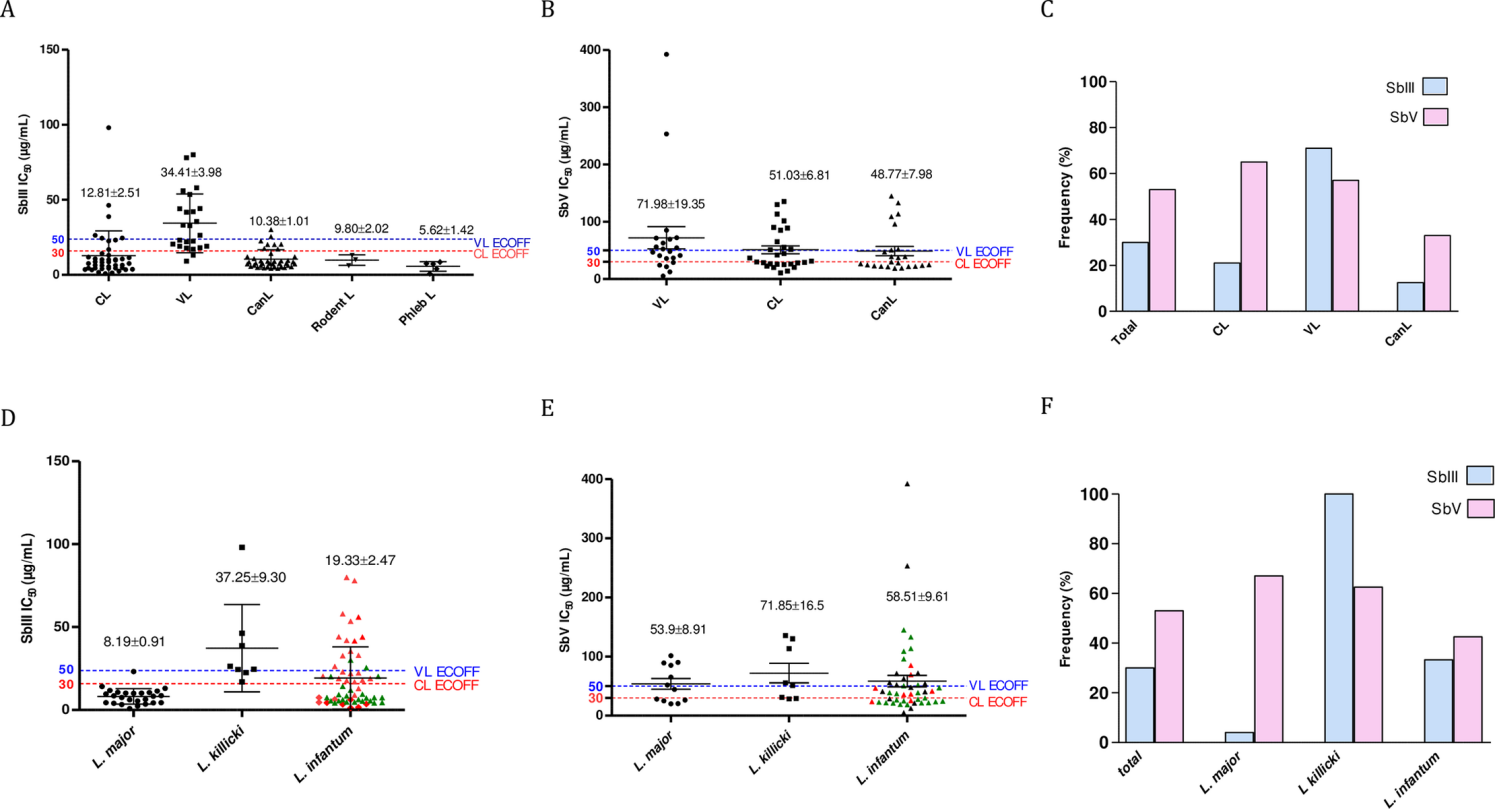
Antimony susceptibility of promastigotes and amastigotes form of *Leishmania* species responsible collected in human (visceral or cutaneous leishmaniasis) in dogs (CanL), rodent (L. Rodent) and sandflies (L. Phleb). SbIII Susceptibility of isolates from human CL and VL, dogs (CanL), rodent (L. Rodent) and sandflies (L. Phleb) (**A**). SbV Susceptibility of isolates from human CL and VL, and dogs (CanL) (**B**). Frequency of S- Leishmania isolates from human CL and VL, and Dogs (CanL) (IC50 above the calculated SbIII or Sb(V)-ECOFF values) (**C**). *In vitro* SbIII susceptibility of *L*. *major*, *L*. *killicki*, *L*. *infantum* parasites. *L*. *infantum* isolated from humans VL(▲) human CL (◆) or dogs (▲) (**D**). *In vitro* SbV Susceptibility of intramacrophagic amastigotes of *L*. *major*, *L*. *killicki* and *L*. *infantum*, from human VL (▲) CL (▲) or Dogs (▲) (**E**). Frequency of S- Leishmania isolates of *L*. *major*, *L*. *killicki* or *L*. *infantum* (IC50 above the calculated SbIII or Sb(V)-ECOFFvalues) (**F**).

Isolates responsible for VL in humans showed significantly lower susceptibility to Sb(III) than those responsible for CL (Fig 5A). *Leishmania* isolates collected from their reservoirs (dogs or rodents) or vectors were mostly highly susceptible to trivalent antimony (Fig 5A, see CanL, Rodent L, Phleb L). Tukey's multiple comparison test revealed highly significant differences in antimony susceptibility between *Leishmania* species (p value <0.0001). We then categorized the isolates as susceptible (S+, IC50 below the ECOFF value) or insusceptible (S-, IC50 above the ECOFF value) according to the previously determined Sb(III)- and Sb(V)-ECOFF values.

**Fig 6:**
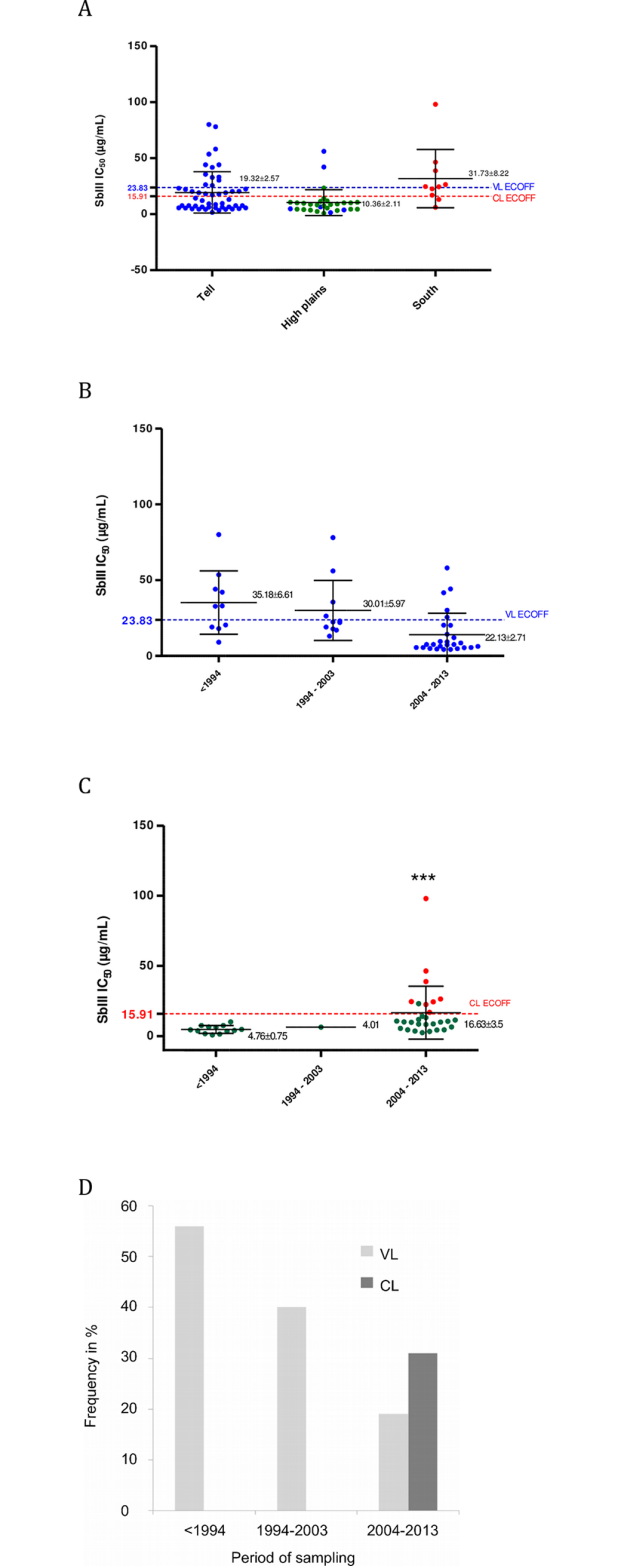
Spatial and temporal analysis of the antimony susceptibility of *Leishmania* isolates from Algeria during 1994 to 2013. Antimony (SbIII) susceptibility of *Leishmania* isolates according to the 3 biogeographic areas (**A**). Antimony (SbIII) susceptibility of *Leishmania* isolates causing VL (**B**) or CL (**C**) according to the three decades, beginning in 1994. Evolution of the frequency of S- phenotype *Leishmania* isolates during the three last decades (**D**). *L*. *infantum* (●), *L major* (●), and *L*. *killicki* (●).

The analysis revealed that approximately 52% (13/25) and 48% (10/21) of the isolates collected from patients suffering from VL were not susceptible to Sb(III) and Sb(V), respectively (Fig 5A, 5B and 5C). For the *L*. *infantum* strains collected from dogs, less than 10% (2/32) were of the S- phenotype for Sb(III), and that proportion reached 26% for Sb(V) (Fig 5A, 5B and 5C). Among the isolates responsible for CL, only 10 of the 43 tested isolates (30%) were S- with regard to Sb(III), exhibiting an IC50 above the determined Sb(III)-ECOFF threshold, while 50% of the isolates (14/28) were S- with regard to Sb(V). Interestingly, all the parasites isolated from an arthropod vector or a rodent host (Fig 6A see LPhleb, LRodent) were S+.

We observed that with an IC50 of 8.19±0.91 μg/mL, *L*. *major* isolates were generally significantly more susceptible (P<0.001) to Sb(III) than were the isolates of *L*. *infantum* (IC50 of 19.33±2.47 μg/mL) or *L*. *killicki* (IC50 of 37.25±9.30 μg/mL) (P<0.001) (Fig 5D). A similar trend was observed in intramacrophagic amastigotes (Fig 5E). Overall, approximately 30% of the isolates fell within the S- category (27/88) with regard to Sb(III) (Fig 5F). This proportion was higher when the classification was performed with the Sb(V)-ECOFF threshold: 53% (39/73). Among the species causing CL, all *L*. *killicki* strains were classified as S- (8/8) with regard to Sb(III) (Fig 5D), while 5/8 (62.5%) fell within the S- category when using the Sb(V)-ECOFF value (Fig 5E). For *L*. *major*, only 6% were S- with regard to Sb(III), but 67% were S- with regard to Sb(V), depicting a fairly poor congruence between the two methods. The reasons underlying these results are unknown and need to be investigated further.

### Analysis of antimony susceptibility over a 30-year period

All the isolates collected in the southern departments were less susceptible to Sb(III) (IC50 of 31.71±8.22 μg/mL) than those isolated from patients residing in the Tell area (19.32 ± 2.57 μg/mL) or in the high plains (10.36 ± 2.11 μg/mL) (p<0.01) (Fig 6A). Most of the isolates from the high plains, where the main foci of transmission are located, are clustered below the Sb(III)-ECOFF value for VL and CL. Only two isolates are S-. In the region of the Tell, a wide variation in Sb(III) susceptibility was recorded, with a mean IC50 of 19.32 ± 2.57 μg/mL (Fig 6A).

In *Leishmania* species causing VL, a general trend of increased susceptibility to Sb(III) has been recorded, with mean IC50 values for Sb(III) of 35.18 ± 6.61 μg/mL for samples collected before 1994, 30.01 ± 5.97 μg/mL for the decade 1994–2003, and 22.13± 2.71 μg/mL for the following decade from 2004 to 2013 (Fig 6B). In contrast, for the isolates causing CL, a decrease in susceptibility to Sb(III) has been recorded with an increasing mean IC50 over the same period of time: 4.76 ± 0.75 μg/mL for isolates sampled before 1994 and 16.63 μg/mL for the decade 2004–2013 (Fig 6C). In this latter decade, isolates displaying a lower susceptibility to Sb(III) emerged: 8 isolates (*L*. *killicki* (syn *L*. *tropica*)) out of 29 (28%) had IC50 values above the Sb(III)-ECOFF threshold.

To summarize, while 50% of the isolates from VL cases collected before 1994 displayed an IC50 value above the Sb(III)-ECOFF value, this proportion decreased to less than 20% in the decade 2004–2013. For CL, before 2004, no isolate displayed an IC50 for Sb(III) above the Sb(III)-ECOFF threshold, but this proportion increased to 30% during the last decade of the study (Fig 6D). An overview of Sb(V) susceptibility of leishmania isolates collected during the 3 decades is given (S3 Fig).

### Isoenzyme polymorphisms and antimony susceptibility

**Fig 7:**
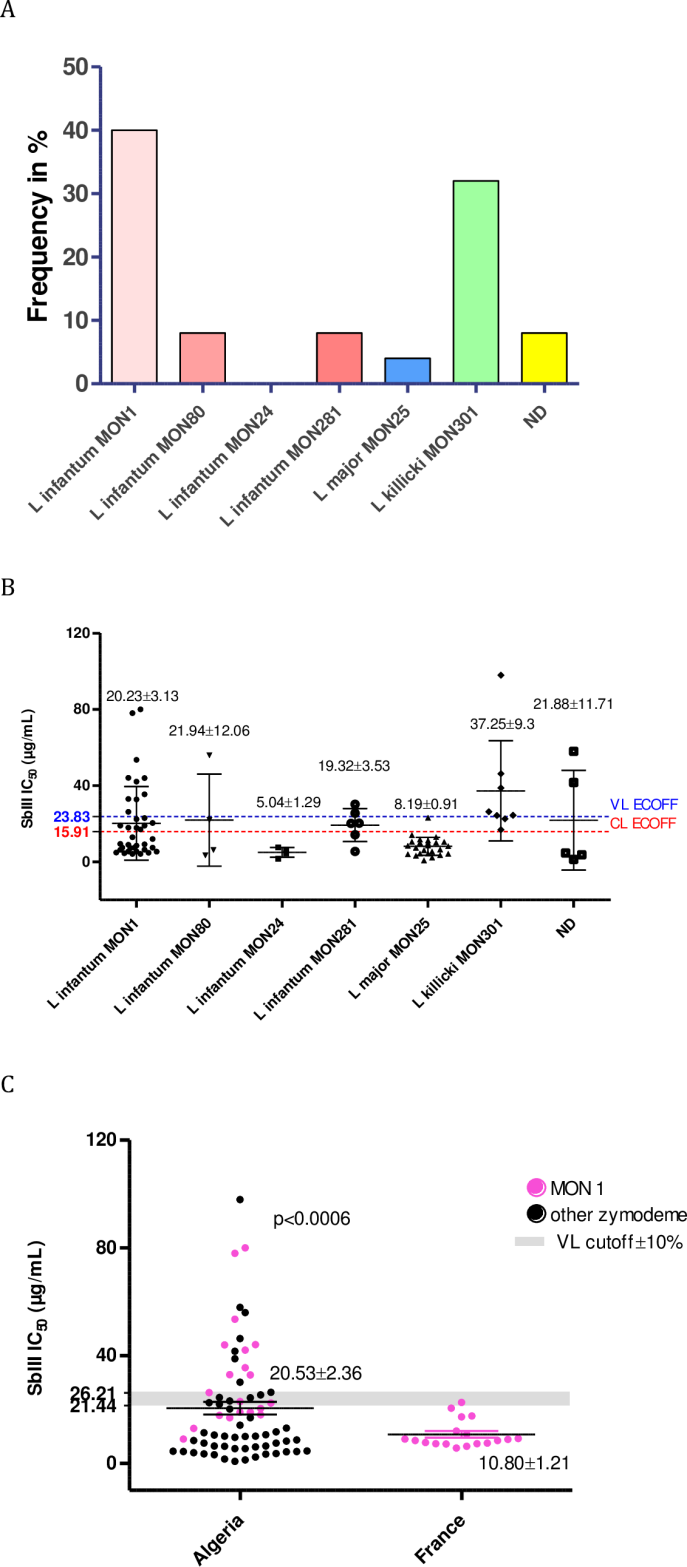
*Leishmania* isoenzymatic polymorphism and antimony susceptibility. Frequency of S- *Leishmania* isolates belonging to various zymodemes known to be transmitted in Algeria (**A**). Antimony (SbIII) susceptibility of *Leishmania* zymodemes in Algeria (**B**). SbIII susceptibility of *Leishmania infantum* MON-1 strains isolated in France and Algeria (**C**).

The analysis of the isoenzymatic polymorphisms of the *Leishmania* populations revealed six zymodemes in Algeria (Table 1). Among the *L*. *infantum* isolates selected in the biobank, 28 ones belonged to the MON-1 zymodeme, 4 to MON-24, 4 to MON-80, and 6 to MON-281 (Fig 7A & 7B). *L*. *major* and *L*. *killicki* (syn *L*. *tropica*) strains belonged to MON-25 and MON-301, respectively. All the isolates characterized as *L*. *killicki* (syn *L*. *tropica*) belonged to MON-301. In addition, 5 isolates could not be identified either at the species or at the zymodeme level, because they did not display a characteristic PCR-RFLP profile or canonical isoenzyme profiles, they were therefore labeled “not determined” (ND). Analysis of variance (ANOVA) revealed a significant association between zymodemes and Sb(III) susceptibility (p<0.01). None of the *L*. *infantum* isolates belonging to the MON-24 zymodeme exhibited IC50 values above the Sb(III)-ECOFF threshold (Fig 7A). However, 25% of the isolates characterized as *L*. *infantum* MON-80 (1/4) exhibited an IC50 value above the computed cutoff value; the corresponding proportions were 33% for *L*. *infantum* MON-1 (10/32) and 40% for *L*. *infantum* MON-281 (2/5). More than 90% (25/26) of the *L*. *major* isolates of the MON-25 zymodeme were classified as S+. Interestingly all the *L*. *killicki* (MON-301) isolates exhibited an IC50 value above the Sb(III)-ECOFF value. Tukey's multiple comparison test revealed a significant difference (P<0.05) in Sb(III) susceptibilities for the *L*. *killicki* MON-301 isolates compared with the other isolates responsible for CL: *L*. *major* (MON-25) and *L*. *infantum* (MON-24) (Fig 7A & 7B).

In the Mediterranean area, *L*. *infantum* is the causative agent of the visceral form of leishmaniosis, affecting both humans and dogs. While in France, canine leishmaniosis was caused only by *L*. *infantum* strains of the MON 1 zymodeme, in Algeria, two zymodemes infected dogs, MON-1 and MON-281. Taking advantage of the large number of *Leishmania* strains and isolates in the collection of the National Reference Centre for Leishmania in Montpellier (France), we selected a set of 17 previously characterized isolates from the Languedoc-Roussillon foci and evaluated their susceptibility to Sb(III). As illustrated in Fig 7C, all French isolates exhibited quite uniform susceptibility to Sb(III) and were S+. They were significantly more susceptible to Sb(III) than those from Algeria (Student’s t-test p<0.001). We noticed that 35% of the tested *L*. *infantum* MON-1 isolates from Algeria were S-.

## Discussion

Leishmaniosis is a globally re-emerging disease in several regions of the world [51,52]. In Algeria, several outbreaks have occurred over the last 30 years, the most significant of which occurred in 2005, when more than 25,000 cases of CL were reported. The population at risk of contracting leishmanioses is large and has increased with the changing demography of the Algerian population during the last 30 years. The incidence of CL in the province of Biskra is significant, with up to 488 cases per 100,000 inhabitants reported between 2004 and 2013. A survey performed in the region of Batna from 2009 to 2014 showed that over 25% of the military personnel present in this area were clinically diagnosed as positive for CL. In Tunisia, VL is also endemic in the northern part of the country, and its incidence shows a general decreasing trend, with 100 cases/year reported between 1996 and 2006 [53]. CL caused by *L*. *major* is a major public health problem in Tunisia. CL occurs mainly in the central and southwestern regions (semi-arid and arid areas), with up to 60% of the population infected in some villages [4,22]. CL caused by *L*. *killicki* (syn. *L*. *tropica*) is also present and is distributed in independent foci [54]. In Morocco, VL is endemic in the Rift and pre-Rift mountains, with over 150 cases per year reported from 2006 to 2008 [4,55]. CL caused by *L*. *major*, which was previously sporadic, has been epidemic since 1976. It occurs in unpredictable outbreaks in the south and the southeast regions of the Atlas Mountains and has recently migrated from the west to the east of the country. In 2001, the Moroccan Health Office reported 2,028 CL cases caused by *L*. *major* and *L*. *tropica*, and 3,414 cases in 2008, indicating a sharp increase in the incidence of leishmanioses in Morocco [4,55]. Among the 3 clinically important *Leishmania* species (*L*. *infantum*, *L*. *major*, *L*. *tropica*), *L*. *tropica* exhibits the widest geographic distribution and is considered a major public health threat.

Currently, the first line of defense against leishmanioses relies on the use of chemotherapy. In Algeria, once they are diagnosed, CL and VL cases are systematically treated. This treatment is delivered free of charge, as recommended by the WHO. Nevertheless, it is estimated that only two-thirds of CL cases are currently treated. The guidelines for the therapeutic protocols for treating patients with CL have been implemented according to the WHO recommendations and published by the Health Office. For all CL, the protocol consists of the administration of 20 mg/kg/day Glucantime for 15 days, via intramuscular injection when multiple lesions are observed or if the lesion is located on the face. For single lesions, the Health Office recommends intradermic (intralesional) administration of 1.5 to 2 mL of Glucantime twice per week for 4 weeks. Nevertheless, alternative treatment strategies are also employed, including the use of oxygenated water either alone or in combination with Glucantime. To treat VL, the Health Office follows the WHO recommendations of intramuscular injections of Glucantime at 20 mg/kg/day for 30 days. Unresponsiveness to treatment and/or relapses are well-documented features in Algeria as well as in the surrounding Mediterranean countries [36,56–60].

The finding that strains with low susceptibility to antimonials are transmitted as well as other strains should drive researchers to conduct studies on the circulation of antimony-resistant strains in Mediterranean countries [39]. The first limitation of such studies lies in the antimony susceptibility test of *Leishmania* parasites. Intracellular amastigotes clearly represent the ideal form of the parasites to be assayed. Unfortunately, methods that involve intracellular amastigotes are labor intensive, difficult to standardize, and dependent upon the nature of the host cell [49,61–64]. The development of reporter gene technologies has enabled the quantification of *Leishmania* parasites in host cells and whole mammalian hosts [65–68], and the capacity of these technologies to determine the drug susceptibility of *Leishmania* field isolates has been tested [56,69]. However, these methods require transfection of the parasites with a reporter gene and the selection of recombinant parasites, which may affect the composition of the isolates [70]. Therefore, their utility for the assessment of *Leishmania* field isolates is limited. Various *Leishmania* species can be grown *in vitro* as amastigotes under axenic conditions [65,71–73]. The determination of drug activity is simple, typically inexpensive and does not require host cells, which makes standardization easier. However, not all *Leishmania* species or strains can be grown in axenic culture conditions. Concerning promastigotes, their usefulness in ascertaining the antimony susceptibility status of *Leishmania* field isolates is a matter of debate. Because these forms are not susceptible to pentavalent antimony, Sb(III) must be used in these tests. Unfortunately, the susceptibility of *Leishmania* promastigotes to Sb(III) does not appear to always reflect the sensitivity of the intramacrophagic forms to pentavalent antimonial formulations [8,74–76]. Interestingly, various experimental studies involving *L*. *tropica* [56] and *L*. *donovani* [77] support the notion that the Sb(III) susceptibility of promastigotes is predictive of the Sb(V) susceptibility of intramacrophagic amastigotes. The present study supports the notion that the Sb(III) susceptibility of promastigotes can be considered predictive of the Sb(V) susceptibility of intramacrophagic amastigotes. Nevertheless, for *L*. *major*, we noticed a bias in the predictive value of the antimony susceptibility of promastigotes. This discrepancy might reflect differences between *Leishmania* strains or species in the activities of reductases involved in the activation of Sb(V). This role is assumed to belong to two reductases, namely, TDR1, a thiol-dependent reductase that belongs to the glutathione S-transferases family and shares homology with the *T*. *cruzi* Tc52 protein [78], and LmACR2, which exhibits homology with the arsenate reductases [79]. However, some variations in the reductase capacity of field *Leishmania* isolates have yet to be investigated. This discrepancy can also reflect the role of some host-derived microbicidal factors involved in Sb(V)-induced intracellular killing of *L*. *major* parasites. Finally, the observed discrepancy may also be related to the greater difficulty of standardizing the intracellular amastigote susceptibility testing system for *L*. *major*.

Antibiotic resistance is currently considered a major threat to human health [80]. In recent years, a global effort to provide a framework aimed at harmonizing the methodologies used to diagnose and monitor antimicrobial susceptibility has been initiated. In Europe, such efforts were first implemented in 1997 by the European Society of Clinical Microbiology and Infectious Disease (ESCMID), which formed the European Committee on Antimicrobial Susceptibility Testing (EUCAST) (http://www.eucast.org/). However, no such efforts have been made for leishmanioses. Because CL and VL constitute a threatening public health concern in Algeria, an effort to survey parasite resistance was launched.

In this analysis, we did not categorize isolates as resistant versus susceptible ones because we did not have documented information about the clinical features in humans and dogs or the Sb-resistant genotypes of isolates. Therefore, the lack of well-defined antimony-resistant *Leishmania* field isolates and the limited number of samples available only allowed us to determine the ECOFF value that defined the threshold between susceptible (S+) and insusceptible (S-) parasite populations, without predicting the presence of truly resistant parasites in the latter. This epidemiological cutoff value could help to clarify the classification of *Leishmania* isolates according to their antimony-susceptibility status. The determination of ECOFF values allows the identification of isolates with low susceptibility that represent a basis for further genetic studies.

The epidemiological cutoff for bacteria is defined by an MIC value that identifies the upper limit for the susceptibility of the wild-type population. Consequently, a microorganism is defined as wild-type if it does not display mutational mechanisms of resistance acquired against a given compound. The IC50 cutoff values that we have determined for *Leishmania* transmitted in Algeria are 23.83 μg/mL and 15.91 μg/mL for isolates responsible for VL and CL, respectively. We therefore propose a first definition for the susceptible phenotype (S+) and insusceptible phenotype (S-) of *Leishmania* isolates. This attempt to define the susceptible population of *Leishmania* will be the first step towards the definition of clinical breakpoints, which will be of interest in investigating more precisely the link between the isolate susceptibility to antimony and the clinical outcomes. Additional studies taking other clinical data into account, particularly the clinical response to treatment, would allow more precise definitions of the clinical breakpoints for the *Leishmania* species responsible for VL and CL.

Based on the ECOFF values defined for *Leishmania* species causing VL and CL, we found that *L*. *killicki (*syn *L*. *tropica)*, which was collected in the endemic CL area of Ghardaia [80], appears to not be susceptible to Sb(III) antimony, and the majority of the isolates (5/8) were also not susceptible to Sb(V). The expansion of these peculiar taxa in Algeria explains the increase in the frequency of the (S-) phenotype detected during the last decade (2004–2013) in our study.

Another striking observation was the global decrease in the frequency of the (S-) phenotype in *Leishmania* strains causing the human VL over the last three decades. This feature must be confirmed with a larger number of isolates available in the biobank of the Pasteur Institute of Algiers.

Interestingly, isoenzyme genotyping presents some predictive value for the identification of the susceptible phenotype. Indeed all *L*. *infantum* strains of the MON-24 zymodeme were (S+), while all *L*. *killicki* (*L*. *tropica*) strains belonging to the MON-301 zymodeme exhibited an (S-) phenotype. It would be interesting to further analyze the antimony susceptibility status of additional *L*. *killicki* MON-301 and *L*. *infantum* MON 24 isolates to strengthen the observations of the current study.

We compared the antimony susceptibility phenotypes of isolates from two geographically distinct regions that differ in terms of the history of antimony consumption: (i) Algiers (Algeria), where there is low antimony pressure in the canine reservoir, and (ii) Cévennes (France), where antimony has been widely used to treat canine leishmaniosis since the 1980s. All isolates belonged to the *L*. *infantum* MON-1 zymodeme. The French isolates appeared to be sensitive to Sb(III) (S+), while 35% of the Algerian isolates were not (S-). These results revealed a significant difference in antimony susceptibility between the two regions. This observation might indicate that drug pressure is not the only underlying factor that triggers the selection of antimony resistant strains in the field [81].

This study is the first one to address the occurrence of the transmission of *Leishmania* isolates with low susceptibility to antimony in Algeria. Leishmanioses are classified by the WHO as neglected tropical diseases, and there are limited resources for chemotherapy. It is therefore of interest to preserve the therapeutic efficiency of antimonials to cure leishmanioses by preventing the emergence of drug-resistant organisms.

## Supporting information

S1 Fig Samples distribution according to the localities of sampling, the clinical forms (for human derived samples) and the decade of sampling. (TIF)

S2 Fig Schematic representation of the three geographic areas of Algeria (left panel) and the total number of cases recorded in these areas over the three consecutive decades (right panel). Data were collected from the Algerian National Public Health Institute (INSP).
(TIF)

S3 Fig Antimony (SbV) susceptibility of *Leishmania* isolates causing VL or CL according to the three decades, beginning in 1994. (TIF)

S1 Table Isolates identification, clinics, year of sampling, *Leishmania* species identification, zymodeme identification, locality of sampling, country of sampling, susceptibility towards SbIII, susceptibility status of promastigotes, susceptibility towards SbV, susceptibility status toward SbV, period of sampling in patients (B: before treatment; A: After treatment). (DOCX)
