# Supplementary figures and images for: Antimony susceptibility of Leishmania isolates collected over a 30-year period in Algeria
Source: PLoS Negl Trop Dis. 2018 Mar 21;12(3):e0006310. doi: 10.1371/journal.pntd.0006310 (PMC5889277; doi:10.1371/journal.pntd.0006310)

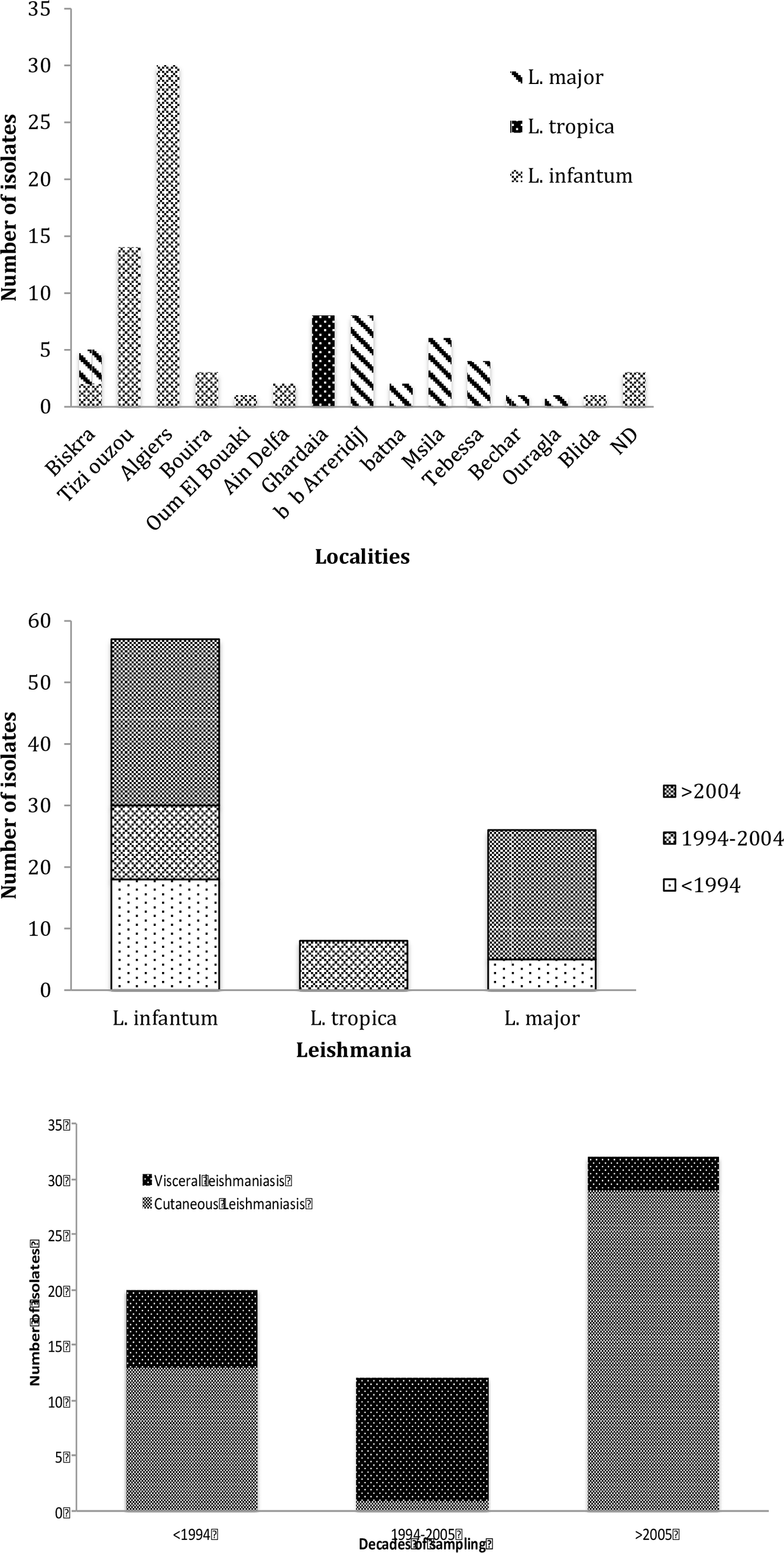

Supplement: S1 Fig — (TIF) [file pntd.0006310.s001.tif]

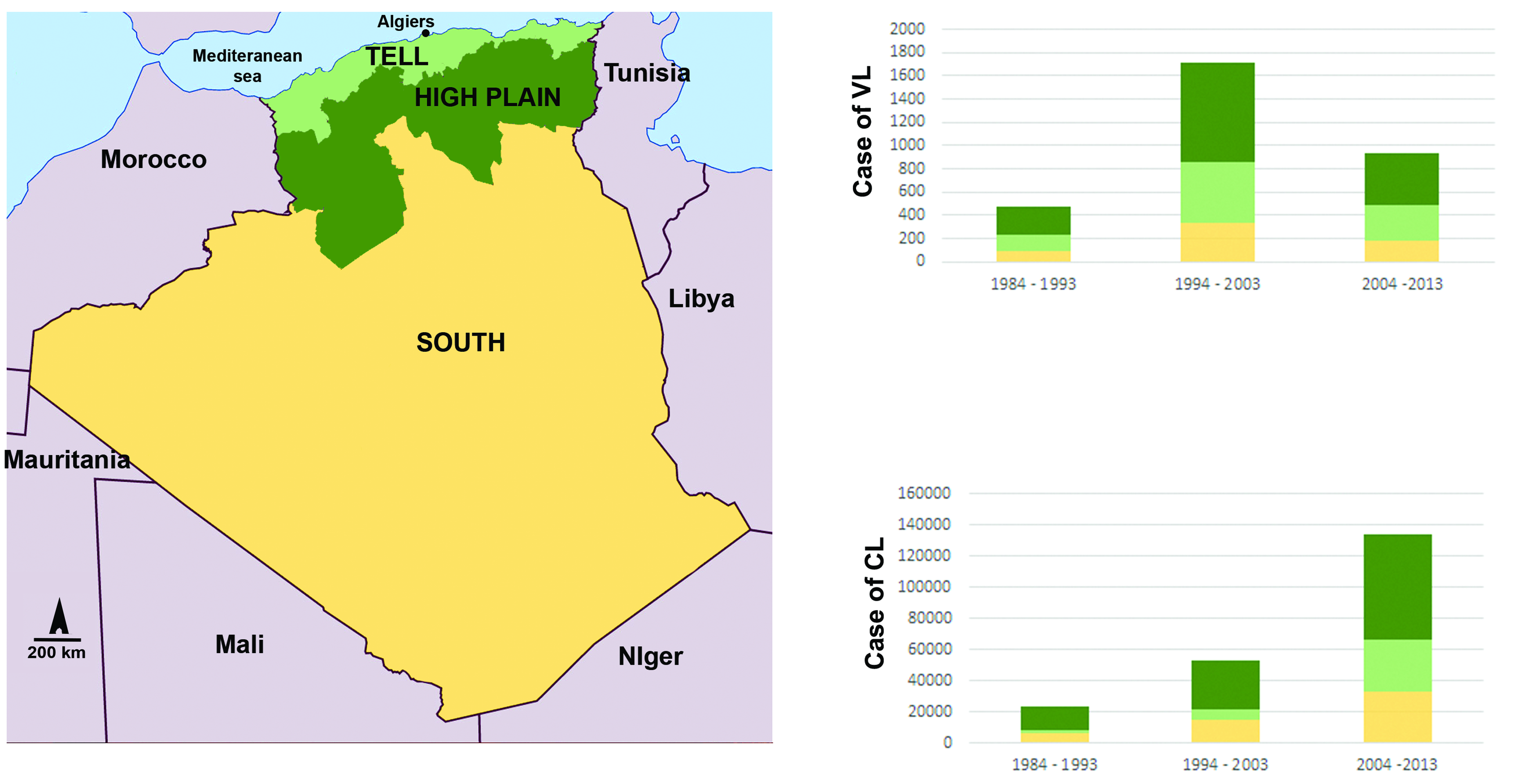

Supplement: S2 Fig — Schematic representation of the three geographic areas of Algeria (left panel) and the total number of cases recorded in these areas over the three consecutive decades (right panel). Data were collected from the Algerian National Public Health Institute (INSP). (TIF) [file pntd.0006310.s002.tif]

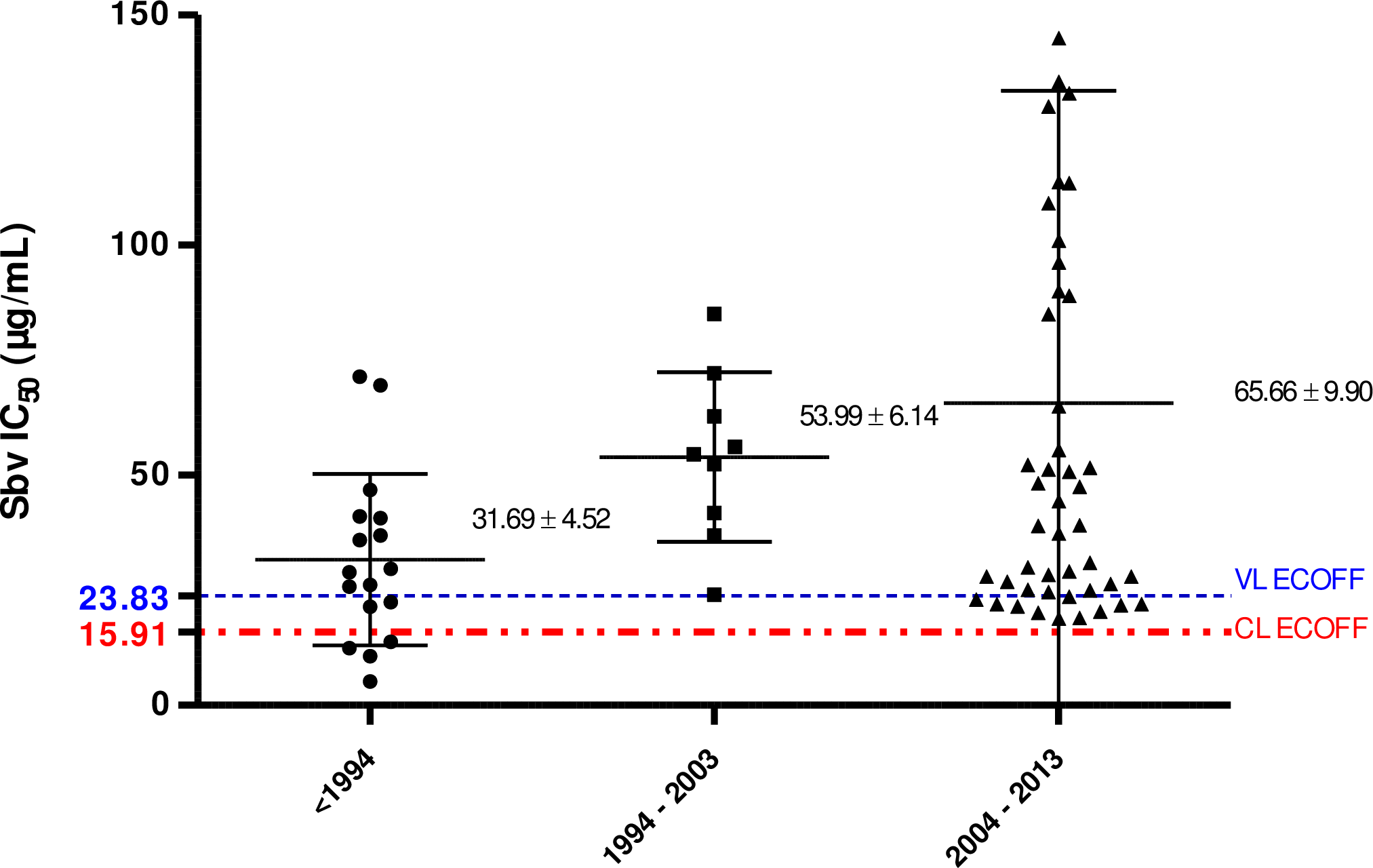

Supplement: S3 Fig — (TIF) [file pntd.0006310.s003.tif]
