# Supplementary material for: Antimony susceptibility of Leishmania isolates collected over a 30-year period in Algeria
Source: PLoS Negl Trop Dis. 2018 Mar 21;12(3):e0006310. doi: 10.1371/journal.pntd.0006310 (PMC5889277; doi:10.1371/journal.pntd.0006310)
Supplement: S1 Table — (DOCX) [file pntd.0006310.s004.docx]

| Isolates | Type | Year | species | Zymodeme | Locality | Country | IC_50_ pro | Phenotype Pro | IC_50_ ama | Phenotype Ama | Chemotherapy  B/A |
| --- | --- | --- | --- | --- | --- | --- | --- | --- | --- | --- | --- |
| MHOM/DZ/1981/LIPA40 | VL | 1981 | *L. infantum* | MON 1 | Biskra | Algeria | 9.00 | S+ | 12.38 | S+ | B |
| MHOM/DZ/1983/LIPA118 | VL | 1983 | *L. infantum* | MON 1 | Tizi Ouzou | Algeria | 18.00 | S+ | 28.91 | S+ | B |
| MHOM/DZ/1987/LIPA170 | VL | 1987 | *L. infantum* | MON 1 | Tizi Ouzou | Algeria | 44.00 | S- | 40.72 | S+ | B |
| MHOM/DZ/1990/LIPA245 | VL | 1990 | *L. infantum* | MON 1 | Biskra | Algeria | 42.12 | S- | 21.39 | S+ | B |
| MHOM/DZ/1987/LIPA177 | VL | 1987 | *L. infantum* | MON 1 | Bouira | Algeria | 80.00 | S- | 71.42 | S- | B |
| MHOM/DZ/1987/LIPA187 | VL | 1987 | *L. infantum* | MON 1 | Tizi Ouzou | Algeria | 19.00 | S+ | 5.19 | S+ | B |
| MHOM/DZ/1990/LIPA277 | VL | 1990 | *L. infantum* | MON 1 | Blida | Algeria | 20.38 | S+ | 41.05 | S+ | B |
| MHOM/DZ/1996/LIPA480 | VL | 1996 | *L. infantum* | MON 1 | Algiers | Algeria | 26.28 | S- | 52.48 | S- | B |
| MHOM/DZ/1996/LIPA469 | VL | 1996 | *L. infantum* | MON 1 | Bouira | Algeria | 35.51 | S- | 72.19 | S- | B |
| MHOM/DZ/2002/LIPA1323 | VL | 2002 | *L. infantum* | MON 1 | Tizi Ouzou | Algeria | 17.86 | S+ | 56.13 | S- | B |
| MHOM/DZ/2001/LIPA1191 | VL | 2001 | *L. infantum* | MON 80 | Algiers | Algeria | 22.00 | S+ | 54.57 | S- | B |
| MHOM/DZ/2002/LIPA1338 | VL | 2002 | *L. infantum* | MON 1 | Bouira | Algeria | 78.00 | S- | 62.77 | S- | B |
| MHOM/DZ/2005/LIPA15/05 | VL | 2005 | *L. infantum* | MON 1 | Tizi Ouzou | Algeria | 44.13 | S- | 48.26 | S- | B |
| MHOM/DZ/1995/LIPA430 | VL | 1995 | *L. infantum* | MON 80 | Oum El Bouaki | Algeria | 56.00 | S- | 24.00 | S+ | B |
| MHOM/DZ/1993/ LIPA390 | VL | 1993 | *L. infantum* | MON 1 | Ain Defla | Algeria | 53.51 | S- | 69.52 | S- | B |
| MHOM/DZ/1988/ LIPA227 | VL | 1988 | *L. infantum* | MON 1 | Algiers | Algeria | 33.00 | S- | 46.84 | S- | B |
| MHOM/DZ/1982/LIPA99 | VL | 1982 | *L. infantum* | MON 1 | Algiers | Algeria | 32.81 | S- | 35.90 | S+ | B |
| MHOM/DZ/1996/ LIPA474 | VL | 1996 | *L infantum* | MON 1 | Algiers | Algeria | 22.46 | S- | 36.89 | S+ | B |
| MHOM/DZ/1999/LIPA979 | VL | 1999 | *L. infantum* | MON 1 | Tizi Ouzou | Algeria | 23.00 | S- | 85.07 | S- | B |
| MHOM/DZ/1999/LIPA979 c1 | VL | 1999 | *L. infantum* | MON 1 | Tizi Ouzou | Algeria | 19.00 | S+ | - | - | B |
| MHOM/DZ/1999/LIPA979 c4 | VL | 1999 | *L. infantum* | MON 1 | Tizi Ouzou | Algeria | 13.00 | S+ | - | - | B |
| MHOM/DZ/1999/LIPA979 c7 | VL | 1999 | *L. infantum* | MON 1 | Tizi Ouzou | Algeria | 17.00 | S+ | - | - | B |
| MHOM/DZ/2010/LIPA155/10 | VL | 2010 | *Leishmania* | ND | Tizi Ouzou | Algeria | 41.67 | S- | 253.50 | S- | B |
| MHOM/DZ/2011/LIPA222/11 | VL | 2011 | *Leishmania* | ND | Tizi Ouzou | Algeria | 58.00 | S- | 392.50 | S- | B |
| MHOM/DZ/1993/LIPA404 | CL | 1993 | *L. infantum* | MON 24 | Ain Defla | Algeria | 4.49 | S+ | - | - | B |
| MHOM/DZ/1992/LIPA364 | CL | 1992 | *L. infantum* | ND | ND | Algeria | 1.34 | S+ | 13.78 | S+ | B |
| MHOM/DZ/1991/LIPA350 | CL | 1991 | *L. infantum* | ND | ND | Algeria | 3.72 | S+ | 26.15 | S+ | B |
| MHOM/DZ/1991/LIPA330 | CL | 1991 | *L. infantum* | MON 24 | Tizi Ouzou | Algeria | 6.49 | S+ | 22.39 | S+ | B |
| MHOM/DZ/1982/LIPA96 | CL | 1982 | *L. infantum* | MON 24 | Tizi Ouzou | Algeria | 7.55 | S+ | 29.68 | S- | B |
| MHOM/DZ/1993/LIPA406 | CL | 1993 | *L. infantum* | ND | ND | Algeria | 4.67 | S+ | 36.88 | S- | B |
| MHOM/DZ/1993/LIPA405 | CL | 1993 | *L. infantum* | MON 24 | Tizi Ouzou | Algeria | 1.65 | S+ | 10.69 | S+ | B |
| MHOM/DZ/1993/LIPA419 | CL | 1993 | *L. infantum* | MON 80 | Algiers | Algeria | 3.45 | S+ | 25.86 | S+ | B |
| MHOM/DZ/1995/LIPA428 | CL | 1995 | *L. infantum* | MON 80 | Bouira | Algeria | 6.31 | S+ | 41.80 | S- | B |
| MHOM/DZ/2005/LIPA07/05 | CL | 2005 | *L. killicki* | MON 301 | Ghardaia | Algeria | 22.59 | S- | 130.20 | S- | B |
| MHOM/DZ/2005/LIPA10/05 | CL | 2005 | *L. killicki* | MON 301 | Ghardaia | Algeria | 46.36 | S- | 135.50 | S- | B |
| MHOM/DZ/2005/LIPA11/05 | CL | 2005 | *L. killicki* | MON 301 | Ghardaia | Algeria | 16.94 | S- | 28.45 | S- | B |
| MHOM/DZ/2005/LIPA14/05 | CL | 2005 | *L. killicki* | MON 301 | Ghardaia | Algeria | 98.00 | S- | 31.11 | S- | B |
| MHOM/DZ/2011/LIPA279/11 | CL | 2011 | *L. killicki* | MON 301 | Ghardaia | Algeria | 24.43 | S- | 55.55 | S- | B |
| MHOM/DZ/2011/LIPA281/11 | CL | 2011 | *L. killicki* | MON 301 | Ghardaia | Algeria | 26.37 | S- | 51.32 | S- | B |
| MHOM/DZ/2011/LIPA282/11 | CL | 2011 | *L. killicki* | MON 301 | Ghardaia | Algeria | 38.83 | S- | 113.50 | S- | B |
| MHOM/DZ/2011/LIPA283/11 | CL | 2011 | *L. killicki* | MON 301 | Ghardaia | Algeria | 24.50 | S- | 29.13 | S- | B |
| MHOM/DZ/2009/LIPA100/09 | CL | 2009 | *L. major* | MON 25 | B B ARRERIDJ | Algeria | 3.59 | S+ | 20.44 | S+ | B |
| MHOM/DZ/2009/LIPA102/09 | CL | 2009 | *L. major* | MON 25 | B B ARRERIDJ | Algeria | 5.47 | S+ | 44.42 | S- | B |
| MHOM/DZ/2009/LIPA103/09 | CL | 2009 | *L. major* | MON 25 | B B ARRERIDJ | Algeria | 6.49 | S+ | - | - | B |
| MHOM/DZ/2010/LIPA134/10 | CL | 2010 | *L. major* | MON 25 | batna | Algeria | 8.27 | S+ | - | - | B |
| MHOM/DZ/2009/LIPA99/09 | CL | 2009 | *L. major* | MON 25 | B B ARRERIDJ | Algeria | 8.55 | S+ | - | - | B |
| MHOM/DZ/2009/LIPA97/09 | CL | 2009 | *L. major* | MON 25 | B B ARRERIDJ | Algeria | 8.69 | S+ | 51.72 | S- | B |
| MHOM/DZ/2009/LIPA104/09 | CL | 2009 | *L. major* | MON 25 | B B ARRERIDJ | Algeria | 9.73 | S+ | 20.21 | S+ | B |
| MHOM/DZ/2010/LIPA138/10 | CL | 2010 | *L. major* | MON 25 | Msila | Algeria | 10.00 | S+ |  |  | B |
| MHOM/DZ/2009/LIPA96/09 | CL | 2009 | *L. major* | MON 25 | B B ARRERIDJ | Algeria | 10.14 | S+ | 26.45 | S+ | B |
| MHOM/DZ/2011/LIPA175/11 | CL | 2011 | *L. major* | MON 25 | Msila | Algeria | 10.63 | S+ | 85.00 | S- | B |
| MHOM/DZ/2009/LIPA135/09 | CL | 2009 | *L. major* | MON 25 | Msila | Algeria | 11.38 | S+ | - | - | B |
| MHOM/DZ/2009/LIPA98/09 | CL | 2009 | *L. major* | MON 25 | B B ARRERIDJ | Algeria | 11.75 | S+ | 25.20 | S+ | B |
| MHOM/DZ/2010/LIPA136/10 | CL | 2010 | *L. major* | MON 25 | Msila | Algeria | 14.16 | S+ | - | - | B |
| MHOM/DZ/2010/LIPA137/10 | CL | 2010 | *L. major* | MON 25 | Msila | Algeria | 23,17 | S- | - | - | B |
| MHOM/DZ/2012/LIPA339/12 | CL | 2012 | *L. major* | MON 25 | Tebessa | Algeria | 4.36 | S+ | 28.00 | S- | B |
| MHOM/DZ/2012/LIPA443/12 | CL | 2012 | *L. major* | MON 25 | Tebessa | Algeria | 2.38 | S+ | 101.00 | S- | B |
| MHOM/DZ/2012/LIPA445/12 | CL | 2012 | *L. major* | MON 25 | batna | Algeria | 4.46 | S+ | 65.00 | S- | B |
| MHOM/DZ/2013/LIPA339R/13 | CL | 2013 | *L. major* | MON 25 | Tebessa | Algeria | 3.25 | S+ |  |  | A |
| MHOM/DZ/2013/LIPA443R/13 | CL | 2013 | *L. major* | MON 25 | Tebessa | Algeria | 4.39 | S+ | 89.00 | S- | A |
| MHOM/DZ/2013/LIPA445R/13 | CL | 2013 | *L. major* | MON 25 | batna | Algeria | 10.51 | S+ | - | - | A |
| MPSA/DZ/1982/LIPA80 | CL | 1982 | *L. major* | MON 25 | biskra | Algeria | 10.15 | S+ | - | - | A |
| MPSA/DZ/1987/LIPA174 | CL | 1987 | *L. major* | MON 25 | bechar | Algeria | 6.14 | S+ | - | - | B |
| MPSA/DZ/2006/LIPA32/06 | CL | 2006 | *L. major* | MON 25 | ouaragla | Algeria | 13.12 | S+ | 90.01 | S- | B |
| MPLB/DZ/1989/LIPA234 | CL | 1989 | *L. major* | MON 25 | Msila | Algeria | 0.80 | S+ | - | - | B |
| MPLB/DZ/1989/LIPA238 | CL | 1989 | *L. major* | MON 25 | Biskra | Algeria | 7.50 | S+ | - | - | B |
| MPLB/DZ/1989/LIPA233 | CL | 1989 | *L. major* | MON 25 | Biskra | Algeria | 4.04 | S+ | - | - | B |
| MCAN/DZ/2006/ENV1(LEM5269) | CanL | 2006 | *L. infantum* | MON 281 | Algiers | Algeria | 30.20 | S- | 145.00 | S- | B |
| MCAN/DZ/2006/ENV2(LEM5270) | CanL | 2006 | *L. infantum* | MON 1 | Algiers | Algeria | 4.25 | S+ | 28.00 | S+ | B |
| MCAN/DZ/2006/ENV3(LEM5271) | CanL | 2006 | *L. infantum* | MON 1 | Algiers | Algeria | 6.40 | S+ | 23.00 | S+ | B |
| MCAN/DZ/2006/ENV5(LEM5284) | CanL | 2006 | *L. infantum* | MON 281 | Algiers | Algeria | 25.50 | S- | 109.20 | S- | B |
| MCAN/DZ/2006/ENV6(LEM5285) | CanL | 2006 | *L. infantum* | MON 281 | Algiers | Algeria | 20.20 | S- | 133.00 | S- | B |
| MCAN/DZ/2006/ENV7(LEM5286) | CanL | 2006 | *L. infantum* | MON 1 | Algiers | Algeria | 5.50 | S+ | 39.00 | S+ | B |
| MCAN/DZ/2006/ENV8(LEM5287) | CanL | 2006 | *L. infantum* | MON 1 | Algiers | Algeria | 7.25 | S+ | 52.30 | S- | B |
| MCAN/DZ/2006/ENV9(LEM5288) | CanL | 2006 | *L. infantum* | MON 1 | Algiers | Algeria | 4.75 | S+ | 47.50 | S- | B |
| MCAN/DZ/2006/ENV10(LEM5289) | CanL | 2006 | *L. infantum* | MON 1 | Algiers | Algeria | 7.50 | S+ | 19.0 | S+ | B |
| MCAN/DZ/2006/ENV11(LEM5300) | CanL | 2006 | *L. infantum* | MON 1 | Algiers | Algeria | 12.00 | S+ | 50.80 | S- | B |
| MCAN/DZ/2007/ENV18(LEM5483) | CanL | 2007 | *L. infantum* | MON 1 | Algiers | Algeria | 7.50 | S+ | 22.10 | S+ | B |
| MCAN/DZ/2007/ENV22(LEM5487) | CanL | 2007 | *L. infantum* | MON 1 | Algiers | Algeria | 5.40 | S+ | 18.90 | S+ | B |
| MCAN/DZ/2007/ENV25(LEM5490) | CanL | 2007 | *L. infantum* | MON 1 | Algiers | Algeria | 4.50 | S+ | 23.70 | S+ | B |
| MCAN/DZ/2007/ENV27(LEM5492) | CanL | 2007 | *L. infantum* | MON 1 | Algiers | Algeria | 4.90 | S+ | 21.60 | S+ | B |
| MCAN/DZ/2007/ENV29(LEM5494) | CanL | 2007 | *L. infantum* | MON 1 | Algiers | Algeria | 4.25 | S+ | 25.10 | S+ | B |
| MCAN/DZ/2008/ENV32(LEM5678) | CanL | 2008 | *L. infantum* | MON 1 | Algiers | Algeria | 9.50 | S+ | 37.40 | S+ | B |
| MCAN/DZ/2008/ENV34(LEM5680) | CanL | 2008 | *L. infantum* | MON 1 | Algiers | Algeria | 5.50 | S+ | 26.90 | S+ | B |
| MCAN/DZ/2008/ENV38(LEM5684) | CanL | 2008 | *L. infantum* | MON 1 | Algiers | Algeria | 8.50 | S+ | 30.10 | S+ | B |
| MCAN/DZ/2008/ENV40(LEM5687) | CanL | 2008 | *L. infantum* | MON 1 | Algiers | Algeria | 6.25 | S+ | 22.00 | S+ | B |
| MCAN/DZ/2008/ENV41(LEM5688) | CanL | 2008 | *L. infantum* | MON 281 | Algiers | Algeria | 5.50 | S+ | 21.80 | S+ | B |
| MCAN/DZ/2008/ENV44(LEM5691) | CanL | 2008 | *L. infantum* | MON 1 | Algiers | Algeria | 7.50 | S+ | 24.70 | S+ | B |
| MCAN/DZ/2008/ENV47(LEM5694) | CanL | 2008 | *L. infantum* | MON 1 | Algiers | Algeria | 9,25 | S+ | 39.20 | S+ | B |
| MCAN/DZ/2008/ENV48(LEM5695) | CanL | 2008 | *L. infantum* | MON 281 | Algiers | Algeria | 14.25 | S+ | 96.30 | S- | B |
| MCAN/DZ/2008/ENV49(LEM5696) | CanL | 2008 | *L. infantum* | MON 281 | Algiers | Algeria | 20.25 | S+ | 113.80 | S- | B |
| MCAN/FR/1974/LEM43 | CanL | 1974 | *L. infantum* | MON 1 | Hérault | France | 17.30 | S+ | - | - | B |
| MCAN/FR/1975/LEM49 | CanL | 1975 | *L. infantum* | MON 1 | Hérault | France | 7.27 | S+ | - | - | B |
| MCAN/FR/1979/LEM104 | CanL | 1979 | *L. infantum* | MON 1 | Hérault | France | 9.26 | S+ | - | - | B |
| MCAN/FR/1980/LEM192 | CanL | 1980 | *L. infantum* | MON 1 | Hérault | France | 7.28 | S+ | - | - | B |
| MCAN/FR/1981/LEM313 | CanL | 1981 | *L. infantum* | MON 1 | Hérault | France | 6.33 | S+ | - | - | B |
| MCAN/FR/1982/LEM387 | CanL | 1982 | *L. infantum* | MON 1 | Hérault | France | 20.49 | S+ | - | - | B |
| MCAN/FR/1984/LEM570 | CanL | 1984 | *L. infantum* | MON 1 | Hérault | France | 12.07 | S+ | - | - | B |
| IPER/FR/1984/LEM576 | CanL | 1984 | *L. infantum* | MON 1 | Hérault | France | 8.53 | S+ | - | - | B |
| IARI/FR/1984/LEM595 | CanL | 1984 | *L. infantum* | MON 1 | Hérault | France | 7.26 | S+ | - | - | B |
| MCAN/FR/1986/LEM851 | CanL | 1986 | *L. infantum* | MON 1 | Hérault | France | 7.51 | S+ | - | - | B |
| MCAN/FR/1987/LEM1092 | CanL | 1987 | *L. infantum* | MON 1 | Hérault | France | 10.83 | S+ | - | - | B |
| MCAN/FR/1990/LEM1925 | CanL | 1990 | *L. infantum* | MON 1 | Hérault | France | 7.75 | S+ | - | - | B |
| MCAN/FR/1995/LEM3030 | CanL | 1995 | *L. infantum* | MON 1 | Hérault | France | 8.95 | S+ | - | - | B |
| MCAN/FR/2006/LEM5198 | CanL | 2006 | *L. infantum* | MON 1 | Hérault | France | 8.54 | S+ | - | - | B |
| MCAN/FR/2006/LEM5221 | CanL | 2006 | *L. infantum* | MON 1 | Hérault | France | 9.02 | S+ | - | - | B |
| MCAN/FR/2006/LEM5227 | CanL | 2006 | *L. infantum* | MON 1 | Hérault | France | 22.69 | S- | - | - | B |
| MCAN/FR/2006/LEM5229 | CanL | 2006 | *L. infantum* | MON 1 | Hérault | France | 17.67 | S+ | - | - | B |
| MCAN/FR/2006/LEM5331 | CanL | 2006 | *L. infantum* | MON 1 | Hérault | France | 5.67 | S+ | - | - | B |
